# US Adults’ Preferences for Public Allocation of a Vaccine for Coronavirus Disease 2019

**DOI:** 10.1001/jamanetworkopen.2020.23020

**Published:** 2020-09-29

**Authors:** Sarah E. Gollust, Brendan Saloner, Robert Hest, Lynn A. Blewett

**Affiliations:** 1 Division of Health Policy and Management, University of Minnesota School of Public Health, Minneapolis; 2 Department of Health Policy and Management, Johns Hopkins Bloomberg School of Public Health, Baltimore, Maryland; 3 State Health Access Data Assistance Center, Division of Health Policy and Management, University of Minnesota School of Public Health, Minneapolis; 4 Health Policy and Management and Director, State Health Access Data Assistance Center, University of Minnesota, School of Public Health Minneapolis

## Abstract

This survey study examines public perception of high priority groups for receipt of an eventual coronavirus disease 2019 vaccine in case of shortage of supply.

## Introduction

A vaccine against severe acute respiratory syndrome coronavirus disease 2 (SARS-CoV-2) will be essential for mitigating the pandemic. However, given global need, demand is expected to exceed supply. When vaccines were limited during the 2009 H1N1 pandemic, the Centers for Disease Control and Prevention developed recommended priority populations based on ethical criteria.^1^ Experts have begun to identify which groups ought to receive priority for a SARS-CoV-2 vaccine, including elderly people, front-line health care workers, and people with existing medical conditions that put them at high risk of severe illness and death.^2,3,4^

Public engagement can contribute to resource allocation decisions. Incorporating public preferences could advance the perceived legitimacy of vaccine allocation guidelines.^4^ This survey study’s objective is to describe the public’s preferences for allocating a SARS-CoV-2 vaccine.

## Methods

The study was determined exempt by the University of Minnesota Institutional Review Board and a waiver of informed consent was granted because data were deidentified and questions posed minimal risk to participants. Data were collected through a module included on the AmeriSpeak Omnibus Survey, fielded by NORC using telephone and internet modes from April 23 to 27, 2020. Respondents were members of AmeriSpeak, a probability-based panel designed to be representative of the US household population. The panel recruitment rate is 34.0%.

The key measure, adapted from H1N1 public engagement activities in Minnesota,^5^ described the potential for SARS-CoV-2 vaccine scarcity and that health authorities may have to set guidelines. The survey question asked: “It is anticipated that in the next 12-18 months, a vaccine for coronavirus will be available. However, at least at first, there may not be enough to go around. Public health authorities must set guidelines about who gets the vaccine first. Please indicate the level of priority that should be given for each of the listed groups.” Respondents indicated which of 8 groups (based on age, health risk, and employment type) should receive high, medium, or low priority.

Descriptive statistics using NORC-provided survey weights generated nationally-representative estimates. Respondents’ preferences were compared using χ^2^ tests by age, race/ethnicity, and self-rated health status, since coronavirus disease 2019 mortality varies by these factors. Race/ethnicity was self-reported and classified as non-Hispanic White, non-Hispanic Black, non-Hispanic other or multiracial (includes non-Hispanic other; non-Hispanic multiracial; and non-Hispanic Asian), and Hispanic. *P* values were 2-sided, and statistical significance was set at .05.

## Results

The survey participation rate was 14.4%, with a final sample of 1007 adults. Among these, 524 (51.4%) were women, 113 (18.1%) were aged 18 to 29 years, 375 (30.7%) were aged 60 years or older, 645 (62.6%) were White, and 170 (20.3%) reported fair or poor health (Table).

**Table.** Public High-Priority Ratings for Coronavirus Disease 2019 Vaccine Allocation by Subpopulation

Group	Total, unweighted No. (weighted %)	Age group				Race/ethnicity					Health status			
		Unweighted No. (weighted %)			*P* value	Unweighted No. (weighted %)				*P* value	Unweighted No. (weighted %)			*P* value
		18-29 y	30-59 y	≥60 y		Non-Hispanic White	Non-Hispanic Black	Non-Hispanic other	Hispanic		Fair or poor	Good	Very good or excellent	
No. (%)	1007	113 (18.1)	519 (51.2)	375 (30.7)		645 (62.6)	171 (12.0)	74 (8.9)	117 (16.5)		170 (20.3)	363 (37.8)	473 (41.9)	
Front-line medical care staff working with patients with coronavirus	937 (91.6)	103 (87.3)	470 (90.3)	364 (96.2)	.06	605 (92.1)	157 (92.5)	67 (89.9)	108 (89.6)	.81	154 (88.6)	335 (90.5)	447 (94.0)	.24
High risk of dying from the coronavirus														
Children (0-18 y old) with serious illness	807 (81.0)	87 (72.7)	400 (79.3)	320 (88.6)	.006	522 (80.8)	133 (81.1)	59 (80.7)	93 (81.6)	.99	134 (81.8)	285 (80.5)	387 (81.0)	.95
People age 65 y and older	799 (80.6)	80 (70.9)	413 (82.0)	306 (84.0)	.03	503 (80.0)	147 (88.2)	56 (82.6)	93 (76.1)	.21	136 (79.5)	290 (81.3)	372 (80.4)	.93
Middle-aged people with serious illness	745 (75.2)	82 (70.7)	377 (74.7)	286 (78.6)	.36	463 (74.0)	140 (84.9)	52 (72.7)	90 (73.9)	.23	128 (72.8)	264 (75.8)	352 (75.7)	.80
Essential workers who interact with the public (postal workers, grocery clerks, etc.)	743 (72.0)	77 (62.4)	362 (69.6)	304 (81.8)	.003	470 (71.5)	127 (73.4)	55 (74.1)	91 (71.9)	.97	133 (81.2)	254 (65.5)	355 (73.4)	.007
People who are pregnant	627 (64.0)	66 (58.2)	306 (61.4)	255 (71.9)	.04	388 (62.2)	119 (74.4)	42 (60.8)	78 (64.9)	.24	112 (72.2)	217 (59.2)	297 (64.2)	.05
Moderate risk of dying from the coronavirus														
Children (0-18 y old)	348 (39.2)	42 (42.3)	176 (40.1)	130 (36.0)	.56	186 (33.6)	77 (50.1)	23 (36.1)	62 (54.2)	.001	67 (42.8)	132 (40.4)	149 (36.5)	.49
Adults (19-64)	242 (29.0)	29 (31.2)	125 (29.9)	88 (26.1)	.62	115 (21.9)	65 (44.1)	15 (26.2)	47 (46.4)	<.001	55 (35.1)	91 (26.7)	96 (28.1)	.28

The Figure demonstrates respondents’ high willingness to allocate vaccine preferentially to front-line medical workers (937 respondents [91.6%] rated them high priority), high-risk children (807 respondents [81.0%] rated them high priority), and high-risk older adults (799 respondents [80.6%] rated them high priority). Respondents also reported priority for middle-aged people with higher risk (745 respondents [75.2%] rated them high priority) and for essential (nonmedical) workers (743 respondents [72.0%] rated them high priority). Fewer respondents reported high priority for pregnant people (627 respondents [64.0%] rated them high priority). While respondents ranked people with moderate mortality risk the lowest, they were more likely to give higher priority to children (348 respondents [39.2%] rated them high priority) than adults (242 respondents [29.0%] rated them high priority). A total of 142 respondents (17.7%) ranked all 8 groups as high priority.

**Figure.**
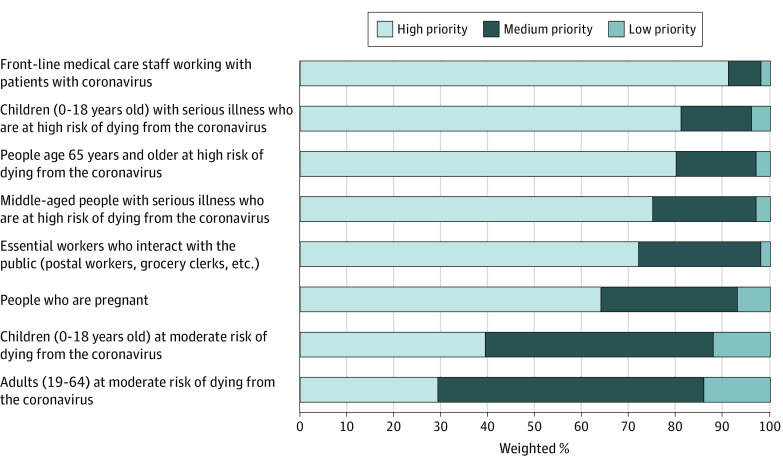
Public Preferences for Coronavirus Disease 2019 Vaccine Allocation Data are from the State Health Access Data Assistance Center coronavirus disease 2019 survey, fielded April 23 to 27, 2020.

Respondents’ age was associated with differences in their likelihood of assigning high priority to children (age 18-29 years: 87 respondents [72.7%]; age 30-29 years: 400 respondents [79.3%]; age ≥60 years: 320 respondents [88.6%]; *P* = .006), people older than 65 (age 18-29 years: 80 respondents [70.9%]; age 30-29 years: 413 respondents [82.0%]; age ≥60 years: 306 respondents [84.0%]; *P* = .03), essential workers (age 18-29 years: 77 respondents [62.4%]; age 30-29 years: 362 respondents [69.6%]; age ≥60 years: 304 respondents [81.8%]; *P* = .003), and pregnant people (age 18-29 years: 66 respondents [58.2%]; age 30-29 years: 306 respondents [61.4%]; age ≥60 years: 255 respondents [71.9%]; *P* = .04) (Table). Respondents’ race and ethnicity was associated with differences in high priority rating for the 2 moderate risk groups, children (White: 186 respondents [33.6%]; Black: 77 respondents [50.1%]; Other: 23 respondents [36.1%]; Hispanic: 62 respondents [54.2); *P* = .001) and adults (White: 115 respondents [21.9%]; Black: 65 respondents [44.1%]; Other: 15 respondents [26.2%]; Hispanic: 47 respondents [46.4%]; *P* < .001). Respondents’ self-reported health status was associated with differences in high priority rating for nonmedical essential workers (fair or poor: 133 respondents [81.2%]; good: 254 respondents [65.5%]; very good or excellent: 355 respondents [73.4%]; *P* = .007) and pregnant people (fair or poor: 112 respondents [72.2%]; good: 217 respondents [59.2%]; very good or excellent: 297 respondents [64.2%]; *P* = .05).

## Discussion

This survey study found that respondents’ preferences were consistent with experts’ emergent recommendations for priority populations for vaccination, suggesting the public would support guidelines that offer vaccine priority to groups defined by age, risk of dying, and employment type.^2,3,4^ More than 90% of respondents identified medical workers as high priority. They also rated people at highest risk of dying as higher priority than people with lower risk.

The study has limitations. The prioritization question did not impose limits on the number of groups respondents could select as high priority. Also, groups assessed did not account explicitly for race/ethnicity or socioeconomic need, but a fuller framework for ethical decision-making should incorporate social justice considerations.^3,6^

Future work on setting priorities for vaccine allocation should use deliberative modes of public engagement to assess public priorities under scarcity and evaluate effective communication. Since the public’s hesitancy toward vaccines is a concern,^2^ consistent and evidence-based communication on the importance of vaccination and the priority groups for receipt of a scarce vaccine is critically needed.
